# The impact of COVID-19 on smoking cessation services with insights for post-pandemic delivery

**DOI:** 10.1371/journal.pone.0295483

**Published:** 2024-09-16

**Authors:** Windi Lameck Marwa, Claire Griffiths, Sophie Edwards, Paul Gately, Caroline Marshall, Carlton Cooke

**Affiliations:** 1 Carnegie School of Sport, Leeds Beckett University, Leeds, United Kingdom; 2 MoreLife, Leeds, United Kingdom; Saint Louis University School of Medicine, UNITED STATES OF AMERICA

## Abstract

Smoking is a leading cause of preventable morbidity and mortality globally. During the COVID-19 pandemic, Smoking Cessation (SC) services faced many challenges, including lockdown and social distancing restrictions. Consequently, SC services had to adapt to the challenges in different ways or halt delivery. This research evaluated the impact of COVID-19 on the delivery and outcomes of SC services. This was achieved by comparing service delivery and outcomes pre-COVID-19 and during the pandemic and drawing insights for the delivery of SC services post-pandemic. Secondary analysis was performed on the data of 11,533 participants who attended the One Life Suffolk (OLS) SC services pre- and during the COVID-19 pandemic. A total of 4923 and 6610 participants attended SC services pre-COVID-19 and during COVID-19 respectively. Fifty-four percent of participants achieved quit status at week-4 while attending the SC services during the COVID-19 pandemic, compared with 46% pre-COVID-19, (*X*^2^(1) = 38.2, *p-*value<0.001). Participants who attended the SC services during the COVID-19 period were 1.7 times more likely to achieve quit status at week-4 than pre-COVID-19. However, the proportion of participants lost-to-follow-up (LTF) was significantly higher during the COVID-19 period (11%) compared to pre-COVID-19 (7%), (*X*^2^(1) = 51.4, *p-*value <0.001). There was an increased participation and quit rate during the pandemic for modified, remotely delivered SC services indicating successful delivery of remote services during the pandemic. Although switching from face-to-face to online helped some smokers to access the service at a time of motivational readiness, despite the COVID-19 restrictions, some smokers could not access or use some aspects of the remote delivery due to a lack of internet access, poor digital literacy, no peer support and no commitment to a group during face-to-face sessions, contributing to an increased rate of LTF. Posing a major challenge to SC services delivery, COVID-19 compelled OLS SC services to adapt and be more innovative in their delivery. SC services need to continue to evolve and adapt by applying the lessons learnt during the pandemic in terms of flexibility and person-centered delivery given what did and did not work well for different demographics within the population.

## Introduction

Smoking is one of the leading causes of preventable morbidity and mortality globally [1, 2]. Several health conditions such as cardiovascular diseases, respiratory conditions and different types of cancers are linked to smoking [3]. Statistics show that, globally, over 8 million individuals die annually due to smoking-related causes [1]. In the USA for instance, between 80–90% of all lung cancer deaths and about 80% of all deaths from chronic obstructive pulmonary diseases (COPD) are attributed to smoking [2] (CDC, 2020). In the UK, nearly three-quarters (72%) of all lung cancer cases are caused by smoking [4]. During the COVID-19 pandemic, smoking was shown to increase the risk of contracting COVID-19 and the development of severe complications [5]. In addition, smoking causes considerable health- and non-health-related costs annually and over a lifetime [6, 7]. The total cost of smoking to society in England for example is estimated to be about £17 billion, including a significant cost to the NHS and social care [8]. Statistics show that in 2019 and 2020 448,031 NHS hospital admissions were related to smoking [9] The cost of smoking-related hospital admissions and primary care treatments amounts to £1.9 billion annually, while local authorities in England spend £1.1 billion every year on care for smoking-related illnesses in later life [8].

**Fig 1:**
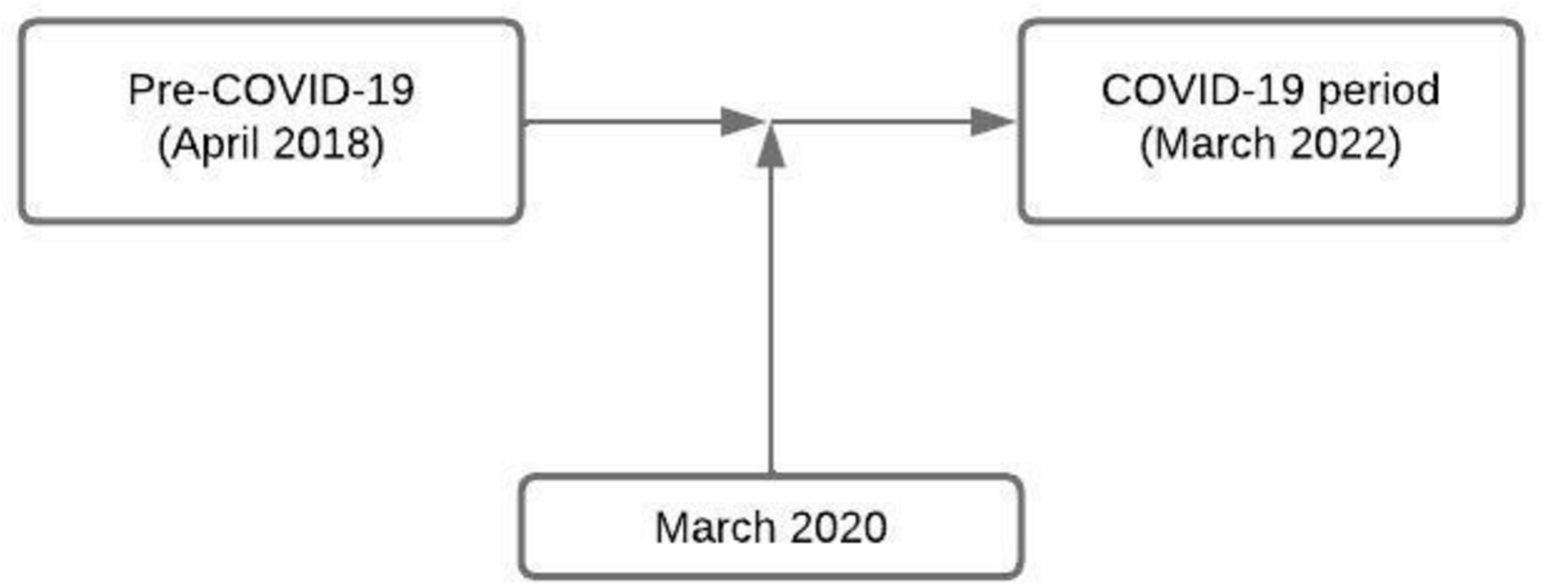
Periods of study.

Quitting smoking has both short- and long-term health and other benefits including reducing the risks of premature death [10, 11] and severe COVID-19 related complications [12]. During the COVID-19 pandemic, smoking cessation (SC) services faced many challenges related to lockdown and social distancing restrictions. Consequently, SC services had to adapt to the challenges in different ways or halt delivery. Adaptations included switching from face-to-face to remote delivery of services and adhering to the requirement to stop carbon monoxide (CO) monitoring from 2020 to 2021 [13]. This research evaluated the impact of COVID-19, the changes adopted during the pandemic and the outcomes of SC services by comparing SC service delivery and outcomes pre- and during the COVID-19 pandemic (Fig 1). In this study, the pre-COVID-19 period started from April 2018 to March 2020 (2 years) and the COVID-19 period started from April 2020 to March 2022 (2 years) (Fig 1). This research uses data collected by OneLife Suffolk on SC services delivered at different settings in the county, including GP, prisons and community centres. Insights from this evaluation for the design and delivery of SC services post-COVID-19 were also considered.

## Material and methods

**Fig 2:**
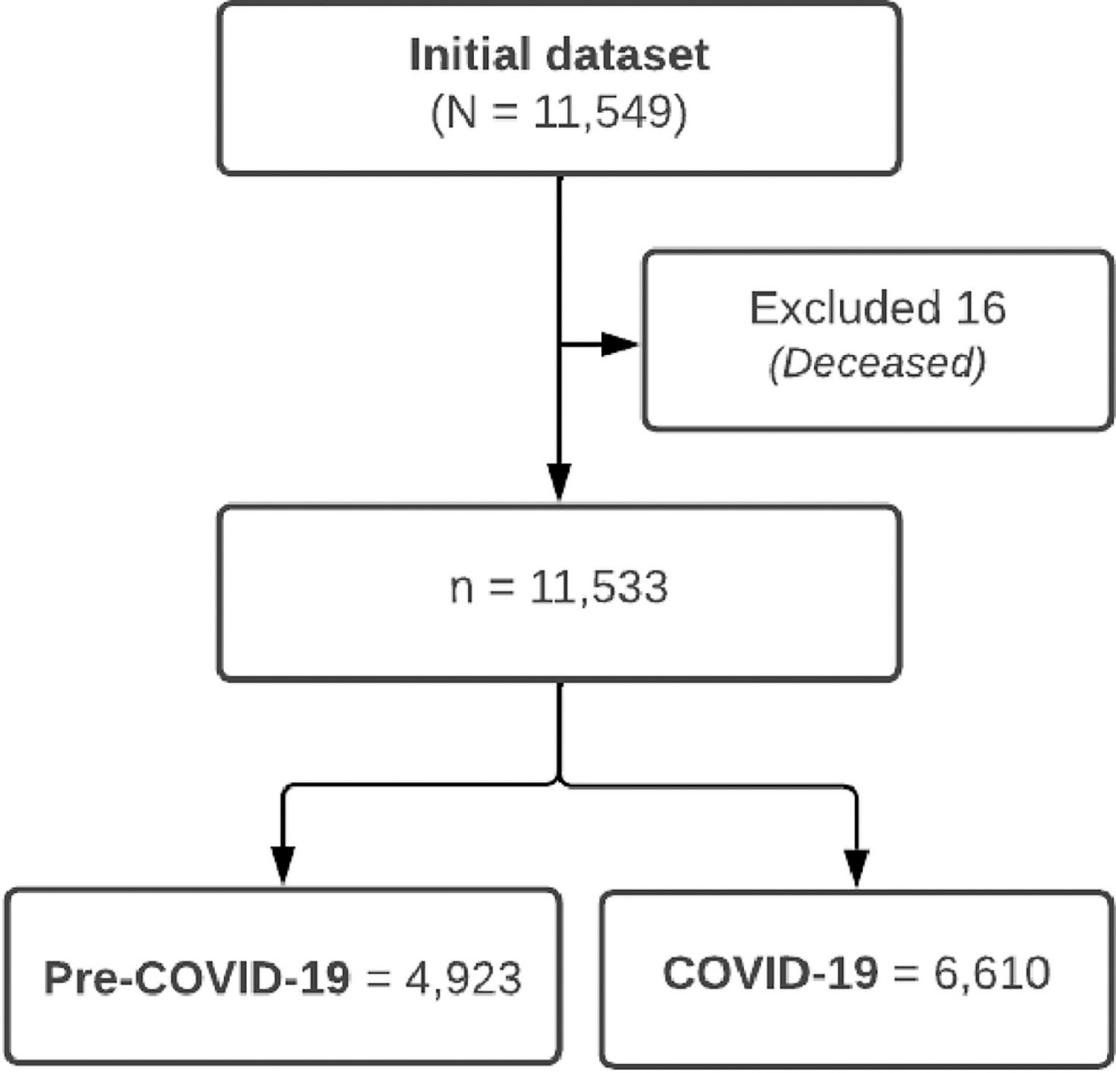
Flowchart of data management.

This study received ethical approval from Leeds Beckett University’s (LBU) Research Ethics Sub-Committee (Approval No. 96676) on 14th April 2022. The researchers analysed an anonymised secondary data set (i.e. individual participants could not be identified) provided by OneLife Suffolk (OLS) for participants who had received SC services. All participants gave consent for their data to be used for research purposes when starting their SC programme with OLS. The researchers accessed the anonymised data set on 12th May 2022. The initial dataset had 11,549 participants, 16 were excluded from the analysis because they were deceased. The final sample was 11,533 participants (Fig 2).

According to the OLS SC programme, participants are considered smokers undergoing treatment and included in service data when they have a four-week quit date set. According to the NCSCT brief, 4-week quit rates, both self-report and CO-verified, have been used to evaluate the effectiveness of SC services since their inception in 1999 [14]). A quit date is vital in determining the effectiveness of treatment, defining the start of the ‘treatment episode’ four weeks prior to the date and from which all other follow-ups are calculated. Four-week quit status can either be self-reported or CO-verified and follow-up must occur 25–42 days from the quit date. Participants who fall outside this period are recorded as Lost-to-follow up (LTF). CO verification is conducted face-to-face but for two years from March 2020, it was suspended [15]. This period coincides with the COVID-19 period referred to in this paper.

The following are definitions of quit status and LTF [16]

- Self-reported 4-week quitter—A treated smoker who reports not smoking for at least days 15–28 of a quit attempt and is followed up 28 days from their quit date (Russell Standard).
- CO-verified 4-week quitter—A treated smoker who reports not smoking for at least days 15–28 of a quit attempt and whose CO reading is assessed 28 days from their quit date and is less than 10 ppm.
- Lost to follow-up (LTF)—A treated smoker who cannot be contacted face to face, via telephone, email, letter or text following three attempts to contact them at different times of day, at four weeks from their quit date (or within 25–42 days of the quit date). Note that the 4-week outcome for this client is unknown and should therefore be recorded as LTF on the monitoring form.

This study used 4-week quit status which is the current standard for measuring the abstinence of clients attending SC services in the UK. According to the NCSC, 4-week quit status provides a good balance between accuracy and practicability [13]. Evidence shows that most smokers relapse within the first few days of a serious quit attempt and the chances of completely quitting smoking increase 5 times in the first 4 weeks [13, 17]. At 4 weeks of smoking cessation, the risk of relapse falls below the likelihood of being a long-term abstainer [13].

A logistic regression was performed to determine the effects of age at quit date, gender, ethnicity, Index of Multiple Deprivation (IMD), occupation, period (pre-COVID-19/COVID-19), Fagerstrom score, sessions attended and total contact time on 4-week quit status. The logistic regression analysis used the data from participants with quit status (i.e. 4-week quit [Yes (n = 6662) or No (n = 3799)]) and excluded those lost-to-follow-up (LTF [n = 1072]). LTF is problematic when it comes to assessing the effectiveness of SC services. In smoking cessation research those LTF are sometimes considered to be smokers, but making such assumptions can lead to an underestimation of treatment effects and imprecision, particularly when the number of LTF is large [18]. In the present study it is not known whether those who are LTF achieved quit status or not.

Nicotine dependence was measured using the Fagerstrom score which ranges from 0–10. A score of less than 4 was considered as less dependence, 4–6 moderate dependence and 7–10 high dependence [19].

All data were analysed using IBM SPSS Statistics for Windows version 28.0 (IBM, Armonk, NY). Multiple Imputations were used in the analyses reported in the results section to optimise power and maintain the sample size by generating 5 imputed datasets based on the observed data (Rubin, 2004; White and Wood, 2011). Independent analyses were conducted on each dataset, then a single estimate was finally generated by pooling the results of each imputed dataset (Rubin, 2004; White and Wood, 2011). A comparison of the results of intention-to-treat (ITT) and imputed data did not show significant differences. Details of the data imputation process, together with comparisons of ITT and imputed data analyses are provided in the S1 File.

### OneLife Suffolk stop smoking service

Commissioned by Suffolk County Council through their Public Health department, OneLife Suffolk (OLS) Smoking Cessation (SC) service provided a free service to the public, supporting smokers in Suffolk to quit smoking. Anyone who had smoked a tobacco product in the past 48 hours and is 12 years old or above was eligible for this service. The programme also supported pregnant and breastfeeding women to quit smoking. The programme ran for 12 weeks providing counselling, treatment (such as Nicotine Replacement Therapy (NRT), Champix (Varenicline), Zyban (Bupropion) and others), and monitored progress using CO levels when permitted. In addition, the OLS SC programme provided tailored behavioural change support to help participants change their behaviour by looking at their current routines and teaching them new coping mechanisms through one-to-one or group sessions. The SC services were delivered in different settings including GP practices, pharmacies, prisons, community centres, hospitals, and maternity wards. The services pre- and during COVID-19 were delivered face-to-face and remotely respectively.

### Changes in delivery of SC services during the COVID-19 pandemic

From March 2020, OLS SC continued to deliver their service, despite the challenges and restrictions due to the COVID-19 pandemic, by adapting to remote smoking cessation consultations and maintaining the provision of stop-smoking medication. However, due to the risk of COVID-19 transmission, from March 2020 CO monitoring in all SC services was paused and all SC service deliveries were, where possible, switched to some form of remote delivery, predominantly online, across the UK [20].

### Changes adopted by the OLS SC programme to adapt to COVID-19 challenges

Following the COVID-19 outbreak, OLS developed a standard operating procedure to describe the different and additional procedures for delivering stop-smoking interventions as part of the OLS Service. Staff training was provided to support the smooth transition from pre-COVID-19 procedures and to help overcome initial concerns/worries. Whilst face-to-face delivery was not possible, appointments were provided via alternative available methods. These included telephone support, e-mail, text, video chat and online groups set up by SC practitioners.

Practitioners continued to follow NCSCT standard treatment programme requirements when delivering these adapted interventions, including the same pharmacological support as during the pre-COVID-19 period. Practitioners agreed and confirmed appointments with participants for a set day and time for the 12-week programme. A contact schedule similar to the one used for face-to-face support was used. The schedule contained pre-quit date, the quit date/quit week, and weekly/fortnightly appointments for at least four weeks post-quit date. Practitioners also made participants aware of the time expectations of each appointment in advance, so they could engage as fully as possible in the remote appointment. Feedback from practitioners identified this as challenging, e.g., as schools were shut, participants were often at home working and home-schooling young children, making it a rather chaotic environment for a participant to fully focus on the remote appointment.

At the start of the delivery of SC services during COVID-19, OLS also set up a ’treatment hub’ to allow for NRT to be delivered to a participant’s home address via courier service within approximately 4 days of the participant’s appointment.

For prescription-only medication, requests for Champix / Zyban were emailed from the NHS mailbox directly to the GP practice. Previously, a letter would have been given to participants at face-to-face appointments for them to hand in at GP practice. When participants accessed remote support services, self-reported smoking status alone, without carbon monoxide (CO) testing, was used for the assessment of non-smoking status. Practitioners therefore needed to be more conscious in their conversation around this to enable an open, honest and truthful account from the participant. Practitioners also used NOPE (not one puff even) to help phrase the question so participants knew they should be reporting even just a puff/drag of a single cigarette.

## Results

### Descriptive statistics

Of the 11,533 participants who participated in the OLS SC programme throughout the pre-COVID and COVID periods that were entered into the analysis, more than half (pre-COVID-19 [57%] and during COVID-19 [62%]) were female and the majority were White (97%). Forty eight percent (pre-COVID-19) and 58% (during COVID-19) were routine/ manual workers and about two-thirds (67% pre-COVID-19 and 61% during COVID-19) were from the 40% most deprived areas of Suffolk. Approximately 10% of all participants were expectant mothers pre- and during the COVID-19 pandemic. The average age of participants in the pre-COVID-19 pandemic period and during the COVID-19 pandemic was 46 ± 16 and 45 ± 15 years respectively, *t* (11531) = 4.31, p = 0.98 (Table 1).

**Table 1:** Participant characteristics.

		Period			
		Pre COVID-19 [n (%)]		COVID-19 [n (%)]	
Programme participation		4923	100	6610	100
Gender	Female	2830	57	4074	62
	Male	2093	43	2536	38
Ethnicity	White	4765	97	6386	97
	Non-White	158	3	224	3
Occupation	Unemployed	1949	40	2369	36
	Routine & Manual	2381	48	3817	58
	Intermediate	253	5	224	3
	Managerial/ Professional	340	7	200	3
IMD quintile	1 (20% most deprived)	2065	42	2454	37
	2	1211	25	1576	24
	3	600	12	977	15
	4	591	12	886	13
	5 (20% least deprived)	456	9	717	11
Pregnant	No	4453	90	5699	86
	Yes	470	10	911	14
Age at quit (years)	Pre-COVID-19: *Mean = 46 ± 16*		Mean Difference = 1.25		*p* = 0.098
	COVID-19: *Mean = 45 ± 15*				

### Participant smoking and intervention characteristics

Fifteen and 13 percent of participants pre- and during COVID-19 had high nicotine dependence respectively. Fifty-nine and 56 percent of participants had smoked for more than 20 years both pre- and during the COVID-19 periods respectively. While the majority of participants (86%) self-referred to the programme pre-COVID-19, only 44% self-referred to the programme during COVID-19. Conversely, less than 1% were provider recruited from the community pre-COVID-19 compared to 44% during the pandemic. The mean Fagerstrom score was 5.44 ± 2.27 and the average number of sessions attended were 5.71 ± 3.54 pre- and 5.8 ± 3.41 during the COVID-19 pandemic (Table 2).

**Table 2:** Participant smoking and intervention characteristics.

		Period (n (%))		
		Pre COVID-19	COVID-19	
Dependence category	Low	2317 (47)	3097 (47)	
	Moderate	1883 (38)	2625 (40)	
	High	723 (15)	888 (13)	
Years smoked	less than 1	278 (6)	156 (2)	
	1—less than 10	585 (12)	932 (14)	
	10—less than 20	1150 (23)	1810 (27)	
	20+	2910 (59)	3712 (56)	
Access Method	Self-referral	4242 (86)	2923 (44)	
	Referral	676 (14)	779 (12)	
	Provider recruited	5 (0)	2908 (44)	
Service provided	One-to-one	4590 (93)	6519 (99)	
	Group	184 (4)	28 (0)	
	Drop-in	149 (3)	0 (0)	
	Telephone	0 (0)	63 (1)	
	Pre-COVID-19	COVID-19		
	Mean ± SD	Mean ± SD	Mean Difference	p-value
Fagerstrom score^$^	5.44 ± 2.27	4.82 ± 2.38	0.62	0.040*
Sessions attended	5.71 ± 3.54	5.80 ± 3.41	-0.09	0.004*
Total contact time	97.55 ± 84.67	93.12 ± 60.50	4.43	<0.001*

^$^ Fagerstrom score indicates nicotine dependence level and ranges from 0–10: Less <4 points, Moderate 4–6 points and High 7–10 points. Pre-COVID-19 (N = 4923) and COVID-19 (N = 6610).

### A comparison of 4-week quit status and LTF pre- and during COVID-19

While 54% of participants who achieved quit status at week 4 attended the SC services during the COVID-19 pandemic, this compared to 46% who attended SC services pre-COVID-19, (*X*^2^ (1) = 38.2, *p-*value <0.001 (Table 3)). However, LTF was significantly higher during the COVID-19 period (11%) compared to the pre-COVID-19 period (7%), *X*^2^ (1) = 51.4, *p-*value <0.001 (Table 3).

**Table 3:** Four week quit status and LTF pre- and during COVID-19.

		Pre COVID-19	COVID-19
4 week quit status	No (n (%))	1511 (40)	2288 (60)
	Yes (n (%))	3065 (46)	3597 (54)
LTF	No (n (%))	4576 (93)	5885 (89)
	Yes (n (%))	347 (7)	725 (11)

Pre COVID-19 63% (i.e. 39% and 24% from Quintile 1 and 2 respectively) of the participants who achieved 4-week quit status were from the 40% most deprived areas. Whereas during COVID-19 60% (i.e. 35% and 25% from Quintile 1 and 2 respectively) of those who achieved 4-week quit status were from the 40% most deprived areas. Also 71% LTF pre-COVID-19 and 62% during the COVID-19 pandemic were from the 40% most deprived areas (Table 4).

**Table 4:** Four-week quit rate differences by IMD and period.

Period	IMD	4 Week quit status		LTF [n (%)]
		No [n (%)]	Yes [n (%)]	
Pre-COVID-19	1 (20% most deprived)	720 (48)	1199 (39)	146 (42)
	2	355 (24)	755 (24)	101 (29)
	3	157 (10)	407 (13)	36 (10)
	4	162 (11)	396 (14)	33 (10)
	5 (20% least deprived)	117 (8)	308 (10)	31 (9)
	Total	1511 (100)	3065 (100)	347 (100)
COVID-19	1 (20% most deprived)	911 (40)	1286 (35)	257 (35)
	2	514 (23)	869 (25)	193 (27)
	3	305 (13)	569 (16)	103 (14)
	4	318 (14)	477 (13)	91 (13)
	5 (20% least deprived)	240 (11)	396 (11)	81 (11)
	Total	2288 (100)	3597 (100)	725 (100)

LFT = Lost-to-follow up

The logistic regression analyses across all participants showed that increased age decreased the odds of achieving week-4 quit status and male participants were 1.2 times more likely to achieve quit status at week-4 than females. Unemployed participants were 0.7 times less likely to achieve 4-week quit status. Participants who attended the SC services during the COVID-19 period were 1.7 times more likely to achieve week-4 quit status than those who attended the services pre-COVID-19. A higher Fagerstrom score was associated with a decreased likelihood of achieving week-4 quit status and the increase in number of sessions (not total contact time) was associated with increased likelihood of achieving quit status in week 4 (Table 5).

**Table 5:** Logistic regression results.

	Categories	Reference	unadjusted OR	95% CI (lower; higher)	adjusted OR	95% CI (lower; higher)
Gender	Male	Female	1.245	1.147, 1.351	1.178	1.060, 1.309
Ethnicity	Non-White	White	0.800	0.641, 0.999*	0.721	0.539, 0.964*
IMD Quintile	*1(20% most deprived)*	*5(20% least deprived)*	0.773	0.670, 0.890	0.985	0.823, 1.179
	*2*		0.948	0.814, 1.103	1.051	0.869, 1.272
	*3*		1.071	0.905, 1.268	1.302	1.055, 1.608
	*4*		0.922	0.779, 1.092	1.128	0.913, 1.394*
Occupation	*Unemployed*	*Managerial*	0.568	0.462. 0.698	0.729	0.563, 0.946*
	*Routine& Manual*		0.875	0.713, 1.073	1.101	0.852, 1.423
	*Intermediate*		1.101	0.824, 1.471	1.232	0.862, 1.762
Period	COVID-19	pre-COVID-9	0.775	0.715, 0.840*	0.638	0.567, 0.718*
Fagerstrom Score			0.921	0.905, 0.937*	0.869	0.849, 0.889*
Access Method	*Referral*	*Self-referral*	1.112	0.979, 1.264	1.057	0.900, 1.241
	*Provider recruitment*		1.275	1.160, 1.401	0.766	0.663, 0.886
Age at quit (years)			1.006	1.003, 1.008*	0.993	0.989, 0.996*
No. of Sessions Attended			1.769	1.728, 1.811	1.850	1.784, 1.918*
Total contact time			1.029	1.028, 1.031	0.999	0.997, 1.001

The logistic regression, *χ*^2^(14) = 4390, p < .001; Nagelkerke’s *R*^*2*^ = 0.47

* indicates significant results (*α*<0.05).

## Discussion

A significantly greater number of participants achieved quit status at week 4 during COVID-19 (3597, 54%) compared to pre-COVID-19 (3065, 46%). Participants who attended the SC services during the COVID-19 period were 1.7 times more likely to achieve quit status at week 4 than pre-COVID-19. However, the proportion of participants LTF was significantly higher during the COVID-19 period (11%) compared to pre-COVID-19 (7%).

There was an increased participation and quit rate during the pandemic for modified, remotely delivered SC services indicating maintenance of successfully delivered services during the pandemic. Although switching from in-person to online helped some smokers to access the service at their time of motivational readiness, despite the COVID-19 restrictions, some smokers could not access or use some aspects of the remote delivery due to a lack of internet access, poor digital literacy, no peer support and no commitment to a group during face-to-face sessions, probably contributing to the increased rate of LTF.

Self-referral to the programme from the community dropped by 38% during the pandemic, whereas the percentage of provider-recruited participants increased by approximately 38% during the pandemic. The higher quit rate during the COVID-19 pandemic could be partly due to either a reliance on self-report during the pandemic or the swift and innovative adaptations adopted by the OLS SC services during the COVID-19 pandemic, or some interaction between these factors. However, the adaptations to service delivery clearly helped the OLS SC services to reach and provide support to those smokers who wanted to quit during the pandemic. Evidence shows that participants who receive good psychological support and pharmaceutical treatment have a higher chance of quitting smoking than those with minimal or no support [21] Furthermore, the COVID-19 pandemic itself could have heightened awareness of the serious effects of smoking on people’s health which may have motivated more smokers to try to quit smoking. Although cigarette sales and consumption increased during the pandemic due to the already existing addiction and possibly some returning to smoking, probably exacerbated by lockdowns, anxiety, boredom and depression leading to unhealthy coping behaviours [22], some studies observed smoking reduction and increased quitting by smokers during this period. For example, a study in Massachusetts USA showed an increased number of both smokers and quitters simultaneously during the pandemic [23]. According to Cunningham [23], smoking during the pandemic could have been perceived as an increased health risk due to COVID-19 and motivated some smokers to quit. Based on this observation, deliberate efforts by the SC services to raise awareness, sensitise and support smokers may well have produced some improvement in the uptake and success rate, in terms of quit status of the SC services during COVID-19.

During the pandemic individuals from deprived areas had higher rates of quitting smoking but LTF increased. One reason that might have contributed to the latter finding could be the switching of services from in-person to online. Although this helped some smokers to access the service at a time of motivational readiness, despite the COVID-19 restrictions, some were unable to access or use some aspects of the remote delivery, including online services due to a lack of access to the internet and poor digital literacy. Also, peer support and commitment to a group during face-to-face sessions that some individuals prefer was not available. A study by Veldhuizen et al. [24], for example, observed a similar pattern and attributed it to the low effectiveness of virtual care compared to in-person care in some populations such as deprived communities. Sanders et al. [25], evaluating Integrated Healthy Lifestyle Services in Suffolk, including the SC services presented here, also showed that a lack of available technological equipment and competence in use were key barriers to initiating and maintaining attendance in online services among vulnerable and disadvantaged people during COVID-19. Such people included Black, Asian or Minority Ethnic groups, routine and manual workers, those living with a learning disability, and high-deprivation groups.

These findings are generalisable in that they add to the evidence that the pandemic exposed further the disparities existing in different sectors of the community through factors such as the digital divide. The digital divide refers to a gap between individuals in the community with full access to digital technologies like the internet and computers and those without [26]. During the COVID-19 pandemic, the digital divide was increased as the internet and digital devices played a significant role in people accessing services, attending medical appointments and staying in touch with their family and friends [26]. As SC services continue to evolve and adapt, consideration of flexible approaches to delivery could help tailor service design and delivery to meet the needs of different groups. Such flexible approaches would include meeting the needs of those who cannot access digital delivery for a variety of reasons and those that prefer face-to-face services, as well as other groups that prefer and benefit from the flexibility of online service delivery which they can more easily fit in around their work-life commitments.

Although the COVID-19 pandemic saw a decrease in self-referral to SC services, which might have been because of the requirement for social distancing and lockdown restrictions, proactive recruitment of participants by practitioners helped the SC programme to increase the levels of participation during the pandemic. This is consistent with a systematic review on recruitment of smokers to SC services, which found that active recruitment of participants is more effective than passive recruitment [27]. Several recruitment strategies have been shown to be more effective than others. For example, a personal phone call, as used multiple times in the adapted SC services, was shown to be more effective than a generic invitation letter (Relative Risk [RR] 40.73, 95% CI 2.53 to 654.74). Tailored messages through an interactive voice response system were also shown to result in a higher recruitment rate than assessment of smoking status alone (RR 8.64, 95% CI 4.41 to 16.93), with the tailored messages also being a feature of the adapted, remote, one to one approach used during COVID-19 [27].

In terms of external validity, the demographics of Suffolk and the focus of the SC service providers does not support generalisability of the findings to all demographic groups and all geographical areas of the UK. However, the findings do inform some areas of generalisability, supported by other research evidence regarding increased health inequalities in deprived areas, which were exacerbated by COVID-19. The majority of the participants (96%) who attended the OLS SC services were White. This is representative of the population of Suffolk which is predominantly White (91.2%) [28]. This will limit the generalisability of the present study to areas of the UK with different demographic characteristics. More females (58% preCOVID-19 and 62% during the pandemic) attended the SC services compared to males. This finding is consistent with the differences in male and female attendance at the NHS SC services in England in 2021 [16]. According to NHS digital [16], for example, more females (57%) did set a quit date compared to males (43%) between April and December 2021.

Forty-eight percent of participants who attended the SC services delivered by OLS pre-COVID-19 and 58% during the pandemic were routine and manual workers. This is more than double their representation in national statistics, again impacting the generalisability of the findings to the UK population but informing the impact of COVID-19 on this demographic. According to the Office for National Statistics, in the UK, around a quarter (23%) of people in routine and manual occupations smoke which is about 2.5 times higher than for people in managerial and professional occupations (9.3%) [29]. In 2019, the prevalence of smoking among people in routine and manual jobs in Suffolk was around five times that of people in other occupations [30]. The prevalence of smoking in Suffolk varies between districts and boroughs, with higher rates in more deprived districts [30]. In 2020 for example, smoking prevalence was highest in Ipswich (20.2%) and lowest in Mid Suffolk (9.5%) [30]. In the current study, about two-thirds of participants (67% pre-COVID-19 and 61% during COVID-19) were from the 40% most deprived areas of Suffolk. According to the OLS SC team, one of their Key Performance Indicators for service delivery was reaching the most deprived population in Suffolk by specifically targeting smokers from those areas. The data for OLS SC services are therefore suggestive of good reach to the population of smokers in the most deprived areas of the county both pre- and during COVID-19. This consistency, which supports the internal validity of this element of the study, also exposes some interesting findings regarding the effects of remote delivery of SC services during COVID-19, where the digital divide could account for an increase in LTF in more deprived areas, leading to increased health inequalities.

The regression analyses in the present study showed that male gender, intermediate occupation, low Fagerstrom score, more sessions attended and attendance during the COVID-19 period increased the chances of achieving a successful quit status, which, apart from the last variable, are all consistent with research evidence for SC interventions, supporting some elements of generalisability of the study findings. Evidence shows that quit rates vary by gender and overall, male smokers are more likely to quit smoking compared to their female counterparts. This is thought to be explained by the fear of weight gain, sex hormones, mood and personality by female smokers [31]. Women tend to gain more weight than men when attempting to quit smoking and those who are older gain more weight than younger smokers [32]. Sex hormones, particularly estradiol and progesterone, are thought to mediate smoking behavior and cessation differences between males and females, with progesterone protecting against nicotine addiction, whereas estradiol enhances vulnerability [33]. A study by Perkins et al. [34] found that women are more likely to increase their puff duration in response to a negative mood compared to men. Also, stress and/or negative affect cues, but not smoking cues, led to significant increases in craving for women compared with men [35].

Fagerstrom score is used to determine the nicotine dependence level [36]. The higher the score the higher the nicotine dependence. Literature shows that Fagerstrom score is an important predictor of smoking cessation [37]. Therefore, ascertaining the level of nicotine addiction of smokers, which is a feature of OLS service delivery, is important to enhance smoking cessation as nicotine dependence is one of the barriers to successful smoking cessation [38].

Quitting smoking without professional help can be challenging. When quitting smoking, smokers have to not only deal with the withdrawal symptoms but also other psychosocial challenges [39]. Thus, the success rate in quitting smoking without professional help is as low as 5% [21]. Combined behavioral and pharmaceutical support increases abstinence rates after 6 months up to 55% [21]. Thus, SC services, such as those delivered by OLS, that provide support to smokers increases the chance of quitting smoking successfully. Evidence indicates that attending more sessions and use of NRT help smokers to cope with withdrawal and psychosocial symptoms, which increases the chance of success in quitting smoking, at least in the short-term, which is consistent with the findings of the present study for delivery of services in Suffolk pre and during the COVID-19 pandemic.

According to OLS SC service standard operating procedures, services provided pre- and during COVID were informed by behaviour change theories and models, and different behavioural change techniques and pharmacological help were used according to individual needs to support smokers to quit smoking (see S2 File for more detail). Identification of the stage of readiness of smokers at the start of their attendance at SC services helped to target support for them to increase their chances of quitting smoking. Smokers who receive good support have a higher chance of quitting smoking than those with minimal or without support [21]. For example, using motivational interviewing techniques, practitioners can help smokers to move from the precontemplation stage through the contemplation stage of the Transtheoretical model to the preparation stage, where plans can be made to initiate NRT when indicated [40]. However, OLS SC services recognise that a significant proportion of quit attempts can be spontaneous. This has led to the COM-B model being used more recently and found to be more appropriate for some smoking cessation clients.

Behavioral counseling techniques are used by OLS to facilitate smoking cessation, improve quit rates and help people maintain quit status. Combined use of behavioral counseling and NRT also significantly improve the smoker’s chance of quitting by minimising the withdrawal symptoms, craving and other psychological challenges [41, 42]. A plan also should be in place to move participants who relapse back to the appropriate stages.

In the current study, the quit status pre-COVID-19 was biochemically verified by CO measurements wherever possible, but during the pandemic this was not possible as CO measurement in all SC services was paused, so quit status during the pandemic period relied on self-reporting [20] providing an unavoidable threat to internal and external validity. This could have had a favourable impact on the overall quit rate during COVID-19 as the quit status could not be objectively verified. However, studies on the validity of self-reporting quit status have reported mixed findings [43]. While some studies have reported a bias on self-reported quit status, especially those in a clinical setting [44, 45], others have shown no difference between the biochemically verified and self-reported quit status, especially where services are offered at the community and population level [46–49] In a study by Wong et al. [49], which used breath CO (BCO, threshold of ≥7 parts per million) to validate self-reported smoking status, a good agreement between self-report and BCO was found (96.0% sensitivity, 93.3% specificity). This suggests that self-reporting could potentially provide sufficiently accurate quit status for community-based services such as those evaluated in this study, but the extent of false positive self-assessments of quit status in the present study was unknown.

Nevertheless, the evidence provided in this study does suggest that the OLS SC services offered during COVID-19 were successful in attracting significant numbers of people who wanted to quit smoking and through their remote service provision were able to support them to do so at a time which matched their motivational preparedness to take action.

## Conclusions

This study showed that SC services were adapted from in-person to remote delivery and successfully supported smokers to quit during the COVID-19 pandemic, including good reach in the most deprived areas of the county. However, there was an increase in LTF, which could be due to a combination of factors such as relapse, or no longer needing support, or where personal circumstances meant participants could not continue during the follow up period. Insights for post pandemic delivery include some positive findings that demonstrate that remote delivery can be effective for some smokers, where they can access support online, with no travel commitments and more flexibility in the timing of when they engage with support. However, remote services do not work for all smokers as some were unable to access or use some aspects of the remote delivery, including online services due to a lack of access to the internet and poor digital literacy. Also, peer support and commitment to a group during in-person sessions that some individuals prefer was not available. Taken together these findings support the need for flexible approaches to SC service delivery where remote, in person or a blended approach will allow all smokers to access the type of service that will help them quit smoking.

### Future considerations

Follow-up qualitative studies involving SC practitioners and participants are needed to explore the range of possible explanations that would provide a better understanding of the factors exposed in this study. Such studies would provide evidence that will further inform service deliverers when adapting service delivery to suit participant circumstances and preferences, whether that is for in-person, blended or remote delivery. Although COVID-19 posed a huge challenge to SC service delivery, it compelled SC services that continued to operate to adapt their delivery and be more innovative in achieving their goals. SC services need to continue to evolve and adapt to changes by applying the lessons learnt during the pandemic in terms of what worked well and what did not for different demographics within the population.

## Supporting information

S1 File (DOCX)

S2 File (DOCX)
