# Supplementary material for: The impact of COVID-19 on smoking cessation services with insights for post-pandemic delivery
Source: PLoS One. 2024 Sep 16;19(9):e0295483. doi: 10.1371/journal.pone.0295483 (PMC11404821; doi:10.1371/journal.pone.0295483)
Supplement: S1 File — (DOCX) [file pone.0295483.s001.docx]

**Supplementary material 1**

**Treatment of Missing Data**

Missing data can be problematic in quantitative research. Missing data must be handled appropriately to prevent the introduction of bias in the data which would distort the interpretation of the findings (Gönülal, 2019; Sterne et al 2009). This supplementary material provides an outline of the approach followed to address the issue of missing data.

*Degree of missingness for each variable of interest*

Seventeen variables of interest were included in the analysis. While 4/17 variables (i.e. age at quit date, total contact time, pregnant, period and gender) had complete data, 13/17 variables had some degree of missingness (Table 1).

Table 1: Overview of missingness

|  | Complete: n (%) | Incomplete: n (%) |
| --- | --- | --- |
| Variables | 4 (24) | 13 (76) |
| Cases | 3,611 (31) | 7,922 (69) |
| Value | 165,758 (90) | 18,770 (10) |

The percentage of missingness ranged from 0.1 - 39.7% (Table 2). This level of missingness was substantially sufficient to warrant an investigation and data imputation.

Table 2: Degree of Missingness by Variables

| Variable | Missing data | |
| --- | --- | --- |
|  | n | % |
| Access method | 4580 | 39.7 |
| Years smoked | 3852 | 33.4 |
| Dependence category | 3275 | 28.4 |
| Fagerstrom score | 3275 | 28.4 |
| 4-week quit status | 1680 | 14.6 |
| IMD quintile | 141 | 1.2 |
| Service provided | 112 | 1.0 |
| Ethnicity | 105 | 0.9 |
| Occupation | 52 | 0.5 |
| Number of sessions attended | 17 | 0.1 |

*Reasons for Missing Data*

According to OneLife Suffolk (OLS) data collection protocol, sociodemographic variables (e.g. age, gender, ethnicity and occupation) were mandatory to be completed by the participants before enrolling on the programme. These variables had either complete data or very few missing values. The programme-specific variables (e.g. Number of sessions attended, Total contact time, Service provided, Years smoked, Fagerstrom score and Access method) were compulsory to be completed by service providers. These variables had the highest missing value in the dataset. The missingness is therefore greater for the service provider rather than participants (Table 2). Based on this observation, it is less likely that cases with missing data differ significantly from those with complete cases, therefore imputation is a plausible method for addressing missing data.

*Pattern of missingness*

The graph in Figure 1 shows positive monotonicity - an increasing pattern (upward steps) of missing data in the lower right part of the graph.


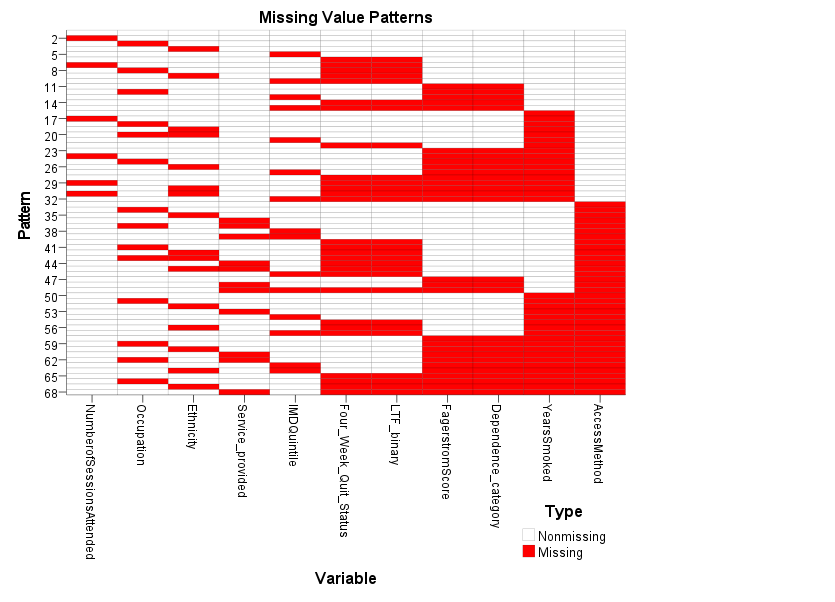


Figure 1: Missing value patterns

Little's test suggested that data were not missing completely at random (MCAR) *χ^2^* (11) 145.8, *p-*value <0.001. Since the missingness could be rationally explained, the data were assumed to be and treated as Missing at Random (MAR).

*Removal of data due to Missing Values*

Before data imputation, data was examined for any influential outliers, invalid measurements, and participants not meeting the inclusion criteria. From the initial dataset of 11,549 participants, 16 were excluded from the analysis because they were deceased. There were neither influential outliers nor invalid measurements. The final sample was 11,533 participants (99.9% of the original sample) (see Figure 1 in the main study).

Several approaches for addressing the issue of missing data were considered. One of the commonly used approaches was list-wise deletion which involves removing the entire record from analysis if a single value is missing (Kang, 2013). If this method was adopted, data of only 31% of participants would be eligible for analysis. This was considered unsuitable since it could significantly reduce the sample size and negatively reduce the statistical power of the analysis (Kang, 2013).

A comparison of participants with complete and incomplete data shows significant differences between them in occupation, IMD quintile, access method, services provided, number of sessions attended and total contact time (Table 3). This suggests that analysis of data of participants with complete data only could lead to biased results, thus justifying a need for data imputation.

Table 3 Differences in Participants with complete and incomplete data (categorical variables)

| Variable | | Complete  n (%) | Incomplete  n (%) | p-value |
| --- | --- | --- | --- | --- |
| Gender | Female | 4739 (60) | 2165 (60) | 0.891 |
|  | Male | 3183 (40) | 1446 (40) |  |
|  |  |  |  |  |
| Occupation | Unemployed | 3040 (38) | 1257 (35) | <0.001 |
|  | Routine & Manual | 4214 (54) | 1957 (54) |  |
|  | Intermediate | 260 (3) | 215 (6) |  |
|  | Managerial/ Professional | 356 (5) | 182 (5) |  |
|  |  |  |  |  |
| IMD Quintile | 1 (20% most deprived) | 3154 (41) | 1315 (36) | <0.001 |
|  | 2 | 1773 (23) | 975 (27) |  |
|  | 3 | 1045 (13) | 508 (14) |  |
|  | 4 | 1009 (13) | 449 (12) |  |
|  | 5 (20% least deprived) | 800 (10) | 364 (10) |  |
|  |  |  |  |  |
| Dependence | Less | 1434 (31) | 1088 (30) | 0.719 |
|  | Moderate | 2406 (52) | 1878 (52) |  |
|  | High | 807 (17) | 645 (18) |  |
|  |  |  |  |  |
| Access Method | Self-referral | 2895 (87) | 2917 (81) | <0.001 |
|  | Referral | 442 (13) | 694 (19) |  |
|  | Provider recruitment | 5 (0.1) | 0 (0) |  |
|  |  |  |  |  |
| Years smoked | less than 1 | 13 (0) | 15 (0) | 0.527 |
|  | 1 - less than 10 | 572 (14) | 544 (15) |  |
|  | 10 - less than 20 | 1094 (27) | 946 (26) |  |
|  | 20+ years | 2391 (59) | 2106 (58) |  |
|  |  |  |  |  |
| Service provided | One-to-one | 7487 (96) | 3540 (98) | <0.001 |
|  | Group | 155 (2) | 29 (1) |  |
|  | Drop-in | 107 (1) | 42 (1) |  |
|  | Telephone | 61 (1) | 0 (0) |  |
|  |  |  |  |  |
| Ethnicity | White | 7544 (96) | 3506 (97) | 0.527 |
|  | Non-White | 273 (4) | 105 (3) |  |
|  |  |  |  |  |
|  |  | *Mean ± SD* | *Mean± SD* |  |
| Age (years) | | 46 ± 15 | 44 ± 15 | 0.775 |
| Fagerstrom score | | 4 ± 2 | 5 ± 2 | 0.598 |
| Number of sessions attended | | 5 ± 4 | 5 ± 3 | <0.001 |
| Total contact time | | 91 ± 77 | 91 ± 53 | <0.001 |

Note that, *X^2^* test and independent sample t-test were conducted for categorical and continuous variables respectively.

Noteworthy, the logistic regression analysis used the data from participants with quit status (i.e. 4-week quit [Yes (n = 6662) or No (n = 3799)]) and excluded those lost-to-follow-up (LTF [n = 1072]). LTF are problematic when it comes to assessing the effectiveness of SC service since it is not known whether those who are LTF achieved quit status or not. Assuming that LFT did not achieve quit status could lead to the underestimation of treatment effects and imprecision in analysis.

*Analysis Used for Missing Data*

Multiple Imputation (MI) was deemed appropriate and used to impute the missing data. Five imputed datasets were generated based on the observed data (White and Wood, 2011). Independent analyses were conducted on each dataset, then a single estimate was finally generated by pooling the results of each imputed dataset (White and Wood, 2011). The Statistical Package for the Social Science (SPSS), version 29 (SPSS INC, Chicago, IL) was used to impute data.

*Number of imputed datasets generated*

Five datasets were imputed using a maximum of 10 iterations with a maximum number of 100 parameters in the imputation model. Models with fewer iterations were completed in sensitivity analysis, but this did not yield differences between the descriptive statistics.

*Variables Included in the Final Imputation Model*

A total of 17 variables were entered into the imputation model which would then be accounted for when imputing missing data. All participant-related variables were included in the MI model to facilitate the most reliable imputation of missing data (Table 4).

Table 4 Variables used in the imputation

| Variable | Variable type |
| --- | --- |
| Access method | Unordered categorical |
| Years smoked | Ordered categorical |
| Dependence category | Ordered categorical |
| Fagerstrom score | Continuous |
| LFT (binary) | Ordered categorical |
| 4-Week Quit Status | Ordered categorical |
| IMD Quintile | Ordered categorical |
| Service provided | Unordered categorical |
| Ethnicity | Unordered categorical |
| Occupation | Ordered categorical |
| Number of sessions attended | Continuous |
| Age at quit date (years) | Continuous |
| Total Contact Time | Continuous |
| Pregnant | Unordered categorical |
| Period | Unordered categorical |
| Gender | Unordered categorical |
| Operational Year | Unordered categorical |

*Comparison of Intention-to-treat and imputed data analyses*

The analyses of intention-to-treat (ITT) and imputed data revealed minor percentage differences in the participant characteristics and week-4 quit status (Table 5 and 6).

Table 5 Descriptive statistics results of ITT and Imputed data analyses

| Variable | | ITT - n (%) | Imputed (pooled) - n (%) |
| --- | --- | --- | --- |
| Gender | Female | 4739 (60) | 6904 (60) |
|  | Male | 3183 (40) | 4629 (40) |
|  |  |  |  |
| Occupation | Unemployed | 3040 (38) | 4318 (37) |
|  | Routine & Manual | 4214 (54) | 6198 (54) |
|  | Intermediate | 260 (3) | 477 (4) |
|  | Managerial/ Professional | 356 (5) | 540 (5) |
|  |  |  |  |
| IMD Quintile | 1 (20% most deprived) | 3154 (41) | 4519 (39) |
|  | 2 | 1773 (23) | 2787 (24) |
|  | 3 | 1045 (13) | 1577 (14) |
|  | 4 | 1009 (13) | 1477 (13) |
|  | 5 (20% least deprived) | 800 (10) | 1173 (10) |
|  |  |  |  |
| Dependence | Less | 1434 (31) | 1088 (30) |
|  | Moderate | 2406 (52) | 1878 (52) |
|  | High | 807 (17) | 645 (18) |
|  |  |  |  |
| Access Method | Self-referral | 2895 (87) | 2917 (81) |
|  | Referral | 442 (13) | 694 (19) |
|  | Provider recruitment | 5 (0.1) | 0 (0) |
|  |  |  |  |
| Years smoked | less than 1 | 13 (0) | 15 (0) |
|  | 1 - less than 10 | 572 (14) | 544 (15) |
|  | 10 - less than 20 | 1094 (27) | 946 (26) |
|  | 20+ years | 2391 (59) | 2106 (58) |
|  |  |  |  |
|  |  |  |  |
| Ethnicity | White | 7544 (96) | 11151 (97) |
|  | Non-White | 273 (4) | 382 (3) |
|  |  |  |  |
| Pregnant | No | 9321 (89) | 10152 (88) |
|  | Yes | 1140 (11) | 1381 (12) |
|  |  |  |  |
|  |  | *Mean ± SD* | *Mean± SD* |
| Age (years) | | 46 ± 15 | 44 ± 15 |
| Fagerstrom score | | 4 ± 2 | 5 ± 2 |
| Number of sessions attended | | 5 ± 4 | 5 ± 3 |
| Total contact time | | 91 ± 77 | 91 ± 53 |

Table 6 Comparison of week-4 quit status for ITT and imputed data analyses

|  | | Imputed - n% | | ITT - n % | | % Difference |
| --- | --- | --- | --- | --- | --- | --- |
| Week-4 quit status | No | 3799 | 32.9 | 3219 | 32.7 | 0.2 |
|  | Yes | 6662 | 57.8 | 5759 | 58.4 | -0.6 |
|  | LTF | 1072 | 9.3 | 875 | 8.9 | 0.4 |

**References:**

Gönülal, T. (2019). Missing Data Management Practices in L2 Research: The Good, The Bad and The Ugly. *Erzincan Üniversitesi Eğitim Fakültesi Dergisi*, 21 (1), 56-73. DOI: 10.17556/erziefd.448559

White IR, Royston P, Wood AM. (2011) Multiple imputation using chained equations: issues and guidance for practice. *Stat Med.* 2011;**30**: 377-399.

Sterne JAC, White IR, Carlin JB, *et al.* (2009) Multiple imputation for missing data in epidemiological and clinical research: potential and pitfalls. *BMJ.* 338.

Kang H. (2013) The prevention and handling of the missing data. Korean J Anaesthesiology. 64(5):402-6. doi: 10.4097/kjae.2013.64.5.402.
