# Supplementary material for: The impact of COVID-19 on smoking cessation services with insights for post-pandemic delivery
Source: PLoS One. 2024 Sep 16;19(9):e0295483. doi: 10.1371/journal.pone.0295483 (PMC11404821; doi:10.1371/journal.pone.0295483)
Supplement: S2 File — (DOCX) [file pone.0295483.s002.docx]

**Supplementary material 2**

**Smoking Cessation Services Support**

1. **Specialist Behaviour Change Support**

OneLife Suffolk (OLS) provides programmes and services to individuals helping them change behaviours and make a difference to their overall health and wellbeing including the stop smoking programme. OLS provides ‘psychologically informed’ practice that offers a ‘middle way’ between narrowly focused standard practice, based on biomedical principles and more therapeutic approaches. Practitioners use a person-centred approach when working with clients and use a variety of psychological models, behaviour change techniques (BCTs) and motivational interview techniques. Through the years OLS has continually developed alongside the evidence, using behaviour change frameworks to provide guiding principles to this.

**Behaviour Change**

OLS utilises BCTs to help clients change their relationship with smoking and other areas of their lives that impact their health including the Transtheoretical Model, COM-B model, self-determination theory, solution-focused theory etc.

**Transtheoretical Model**

The Transtheoretical model provides useful tools and framework when working with some clients. Historically this was the go-to model for smoking cessation, however, over recent years it has been found to have some flaws when looking at smoking cessation in practice. The suggestion that an individual needs to go through pre-contemplation, contemplation, and preparation before taking action is not always seen to be the case. Research shows that around half of all quit attempts in the GP setting are spontaneous, so no planning or preparation at all, just straight to action. This has led to the COM-B model being used more recently and found to be more appropriate for some smoking cessation clients.

**COM-B Model**

The ‘COM-B’ behaviour change model identifies three factors that need to be present for any behaviour to occur: capability, opportunity, and motivation. For smoking cessation to happen, smokers need to have the capability, opportunity and motivation to change. OLS practitioners used the COM-B model to underpin both the intervention and support offered, therefore much of what OLS does is structured in ways to achieve these principles so people can sustain their behaviour change.

**Theory of Planned Behaviour**

Another model that is used in SC is the theory of planned behaviour which links beliefs to behaviours. Based on this theory, three main components- attitude, subjective norms and perceived behavioural control- shape behaviours.

**Solution-focused theory**

OLS also uses solution-focused theory, which as the name says, is focused on supporting clients to look forward and find solutions rather than looking backwards and being focused on problems. It aligns well with our other frameworks as it focuses on growth, self-development and responsibility. Helping clients set goals, think about what life would be like when they achieve some of their goals and how do they keep working towards their goals using their strengths to overcome barriers and continually developing their resilience.

**Self-determination theory**

Self-determination theory (SDT) is a general theory of human motivation that emphasises the extent to which behaviours are relatively autonomous versus relatively controlled.

**Motivational interviewing**

Motivational interview (MI) techniques are also used to structure conversations in a nondirective way to help clients resolve ambivalence (mixed feelings) they may have regarding change. Active listening skills are key to MI and allow Practitioners to really understand the context of the behaviour rather than providing advice prematurely that may not apply to the client’s situation.

Providing affirmations is another MI technique that is used to create rapport with the client and draw out the real strengths that they possess which increases self-efficacy more effectively than simply praising them for ‘good’ behaviour. Positive affirmations focus on specific behaviours or attributes a client has displayed and should be pitched at the right level.

1. **Therapeutic support**

Whilst some of our clients respond well to BCTs and a bit of MI, some have more ingrained thought and emotion patterns that may need to be explored to make changes. To help with discussion of these topics, practitioners use activities from Cognitive Behavioural Therapy (CBT) and Acceptance and Commitment Therapy (ACT).

Using CBT, practitioners can help clients to identify negative thinking patterns and explore how these have formed. This approach helps practitioners to guide clients to challenge the negative thoughts which lessens their impact on emotions and behaviours in the long term. Clients can also explore how their emotions impact their behaviour, for example in emotional smoking, practitioners discuss techniques aimed at managing emotions and finding more positive ways of reacting to them during the sessions.

ACT is used to help clients have a broader understanding of the biopsychosocial model and equip them with the skills that they need to deal with controllable and uncontrollable impacts upon them. Other techniques that are useful for clients to explore include mindfulness techniques which have been shown to help reduce stress and manage cravings.

**The Stop Smoking Programme**

NICE guidance recommends that stop-smoking interventions include both behavioural support and pharmacotherapy/vapes. These should be multi-sessional and tailored to the individual. The way OLS sessions are structured supports behaviour change, and the content of the sessions is informed by the theories and BCTs discussed above, and are built into each session including:

- Goal setting and review of goals
- Action planning
- Barrier identification and problem-solving
- Pros and cons of quitting
- Self-monitoring of behaviour
- Feedback on performance
- Instruction on how to perform behaviour when appropriate
- Utilising social support

Goal setting remains one of the most powerful tools for change. Helping individuals set SMART goals at the end of each session means they can clarify their ideas, focus their efforts, use time and resources productively, and increase their chances of achieving what they want.

Evidence suggests that the top behaviour change techniques within smoking cessation include:

- CO monitoring
- Stop smoking medications
- Self-regulation (barriers, cues, cravings, withdrawal)
- Prompting commitment
- NOPE
- Identity
- Building rapport

Although much of the session content is pre-determined by the NCSCT standard treatment programme, Practitioners still have a lot of influence over how sessions are delivered. By understanding the theories described earlier, Practitioners have a good understanding of how to decide which behaviour change techniques to use and when, as well as which areas to focus on depending on individual groups or individual clients’ needs and barriers.

**Session Overview (Stop smoking curriculum)**

The Stop Smoking curriculum acts as a guide for Practitioners to ensure best practice and high-quality care throughout a client’s journey. Whether the delivery is done one-to-one face-to-face, group, drop-in, on-line or via telephone support, OLS work to a standardised programme of care in line with the National Centre of Smoking Cessation and Training (NCSCT) standard treatment programme (STP). However, the support programme recognises diversity is individualised, and tailored to the client’s needs e.g., pregnant women, young people, and clients with mental health disorders.

1. **Stop Smoking Treatments**

NICE guidance (NG209) recommends that people who want to give up smoking are made aware of the treatment options available and how to access them. The most successful tools to stop smoking include i.) A combination of short-acting and long-acting NRT, ii.) Varenicline (Champix) and iii.) Nicotine-containing vapes (e-cigarettes).
